# Supplementary material for: Exploring the Social Determinants of Mental Health in Colombian Young Adults
Source: Eur J Investig Health Psychol Educ. 2025 Jul 13;15(7):133. doi: 10.3390/ejihpe15070133 (PMC12294809; doi:10.3390/ejihpe15070133)
Supplement: Supplementary file 1 [file ejihpe-15-00133-s001.zip › ejihpe-3723070-supplementary.pdf]

Supplementary Material 1

Exploring the Social Determinants of Mental Health in Colombian Young Adults

Table 1

Descriptive statistics and reliability estimates of the intermediate factors.

	Factor	Items	Scale score	Minimum and maximum scores	Women (*n*= 576)		Men (*n*= 656)		α	ω
					M	SD	*M*	SD		
**Well-being in the environment**	Well-being at home	4	0-6	0-24	17.44	3.70	17.54	3.77	.71	.71
	Well-being in the neighborhood	4	0-6	0-24	13.72	4.89	13.80	4.93	.59	.63
	Well-being in religious organizations	4	0-6	0-24	7.02	7.61	6.26	7.65	.87	.88
	Social welfare, culture and recreation	4	0-6	0-24	8.78	8.64	9.66	8.81	.90	.92
	School wellness	4	0-6	0-24	16.74	3.67	17.22	3.50	.38	.39
	Well-being at work	4	0-6	0-24	16.50	3.97	16.44	4.10	.92	.93
**Protective factors**	Expectation of social support	8	0-6	0-48	25.79	9.08	26.36	9.16	.67	.69
	Healthy lifestyle habits	8	1-6	1-48	25.15	5.88	25.64	6.11	.71	.69
	Psychosocial skills for life	10	1-5	10-50	36.19	7.91	36.34	7.88	.90	.90
**Risk factors**	Worry and emotional distress	5	0-4	0-20	10.82	3.75	10.54	3.545	.66	.67
	Suicidal behavior	3	0-1	0-3	1.04	1.03	0.97	1.05	.63	.63
	Drug use	5	0-4	0-20	2.70	2.68	2.78	2.61	.63	.62
	Conflicts due to drug use	7	0-2	0-14	4.37	3.52	4.73	3.56	.93	.93
	Problematic internet use	4	0-4	0-16	6.27	2.62	6.73	2.77	.45	.55
	Problems due to problematic internet use	7	0-2	0-14	6.31	2.805	6.20	2.94	.85	.85
**Well-being and discomfort in the last week and the last six months**	Emotional symptoms in the last week	8	1-5	8-40	24.08	8.19	23.72	7.86	.91	.91
	Physical symptoms in the last week	5	1-5	5-25	14.03	5.31	13.60	4.91	.82	.82
	Well-being in the last week	4	1-4	4-20	12.49	3.52	12.93	3.54	.78	.79
	Emotional symptoms in the past six months	8	1-5	8-40	23.43	8.17	23.86	8.49	.94	.93
	Physical symptoms in the past six months	5	1-5	5-25	13.72	5.5	13.37	5.30	.88	.88
	Well-being in the last six months	4	1-4	4-20	12.65	3.81	12.85	3.86	.85	.85

Table 2

Spearman correlations between the intermediate determinants among participants without a mental health diagnosis (N=980).

	Variable	1	2	3	4	5	6	7	8	9	10	11	12	13	14	15	16	17	18	19	20	21
1	Well-being at home	—																				
2	Well-being in the neighborhood	.38**	—																			
3	Well-being in religious organizations	.17**	.18**	—																		
4	Social welfare, culture and recreation	.12**	.27**	.34**	—																	
5	School wellness	.25**	.35**	.09**	.20**	—																
6	Well-being at work	.07*	.12**	.25**	.24**	.06*	—															
7	Expectation of social support	.23**	.31**	.23**	.25**	.23**	.24**	—														
8	Healthy lifestyle habits	.23**	.26**	.21**	.36**	.24**	.07*	.22**	—													
9	Psychosocial skills for life	.22**	.20**	.09**	.16**	.18**	.09**	.21**	.31**	—												
10	Worry and emotional distress	-.32**	-.12**	-0.03	-0.01	-.12**	0.01	-0.04	-.26**	-.28**	—											
11	Suicidal behavior	-.25**	-.11**	-0.05	-.07*	-.06*	-0.01	-.06*	-.14**	-.18**	.24**	—										
12	Drug use	-.10**	0.04	-.10**	.07*	0.04	0.05	0.03	0.001	-.10**	.12**	.09**	—									
13	Conflicts due to drug use	-.12**	.06*	-0.05	.07*	0.03	.13**	0.06	0.04	-.07*	.16**	.14**	.51**	—								
14	Problematic internet use	-0.03	.06*	-.11**	0.05	0.03	-0.01	0.02	.13**	0.05	-0.02	-0.01	.31**	.22**	—							
15	Problems due to problematic internet use	-.16**	-0.03	-0.02	0.01	-0.001	.11**	.07*	0.03	-.08**	.24**	.12**	.23**	.47**	.25**	__						
16	Emotional symptoms in the last week	-.28**	-.16**	-0.01	-0.04	-.16**	-0.02	-.07*	-.27**	-.23**	.41**	.33**	.11**	.12**	-0.03	.21**	__					
17	Physical symptoms in the last week	-.20**	-.10**	0.03	0.02	-.11**	0.02	0.009	-.20**	-.12**	.35**	.26**	.09**	.09**	-.09**	.14**	.21**	__				
18	Well-being in the last week	.18**	.14**	0.02	.07*	.19**	0.02	.14**	.27**	.34**	-.13**	-.10**	0.005	0.05	.16**	0.01	.14**	-0.001	__			
19	Emotional symptoms in the past six months	-.25**	-.10**	-0.006	-0.05	-.12**	-0.005	-0.04	-.24**	-.22**	.36**	.33**	.10**	.12**	-0.01	.20**	0.01	.57**	-.09**	__		
20	Physical symptoms in the past six months	-.18**	-.10**	0.02	-0.01	-.07*	0.01	0.006	-.19**	-.12**	.31**	.22**	.08**	.11**	-.07*	.17**	.20**	.73**	-0.05	.70**	__	
21	Well-being in the last six months	.12**	.09**	0.01	-0.01	.12**	-0.02	.12**	.15**	.24**	-.08**	-.07*	0.005	0.02	.09**	0.02	.17**	0.03	.60**	.10**	.17**	__

*$p < .05$; **$p < .01$

Table 3

Spearman correlations between the intermediate determinants among participants with a mental health diagnosis (N=252).

	Variable	1	2	3	4	5	6	7	8	9	10	11	12	13	14	15	16	17	18	19	20	21
1	Well-being at home	—																				
2	Well-being in the neighborhood	.32**	—																			
3	Well-being in religious organizations	.15*	.21**	—																		
4	Social welfare, culture and recreation	.19**	.15*	.27**	—																	
5	School wellness	.23**	.31**	-0.06	.12*	—																
6	Well-being at work	0.04	0.09	.34**	.32**	0.08	—															
7	Expectation of social support	.26**	.26**	.26**	.19**	.13*	.29**	—														
8	Healthy lifestyle habits	.33**	.18**	.24**	.45**	.17**	.27**	.26**	—													
9	Psychosocial skills for life	.26**	.20**	.12*	.23**	.23**	.20**	.13*	.44**	—												
10	Worry and emotional distress	-.35**	-0.09	0.04	-0.09	-0.11	-0.10	-0.08	-.29**	-.24**	—											
11	Suicidal behavior	-.22**	-.15*	-0.07	-0.01	-.16*	0.04	-0.06	-.28**	-.23**	.26**	—										
12	Drug use	-0.04	0.08	-0.08	0.06	0.05	0.08	0.02	0.11	0.06	0.02	0.08	—									
13	Conflicts due to drug use	-0.08	0.05	-0.07	0.06	0.04	.12*	0.09	0.08	0.07	0.11	0.03	.50**	—								
14	Problematic internet use	0.05	0.02	-0.08	0.05	0.07	-0.03	-0.03	0.10	0.06	-.13*	-0.09	.15*	-0.007	—							
15	Problems due to problematic internet use	-0.11	0.04	-0.01	0.02	0.07	.15*	0.08	0.04	0.007	.17**	0.07	.20**	.41**	0.08	__						
16	Emotional symptoms in the last week	-.34**	-.19**	-0.11	-0.03	-.25**	-0.05	-0.07	-.31**	-.25**	.38**	.42**	0.11	0.09	-0.03	.21**	__					
17	Physical symptoms in the last week	-.14*	-0.01	-0.01	-0.02	-0.08	0.09	0.05	-.18**	-0.06	.37**	.30**	0.10	0.09	-.15*	.25**	.21**	__				
18	Well-being in the last week	.25**	.18**	.16*	.18**	.18**	.18**	.18**	.36**	.41**	-.24**	-.31**	.13*	.13*	.26**	0.04	.25**	-.15*	__			
19	Emotional symptoms in the past six months	-.24**	-.16**	-.15*	-0.04	-.15*	-0.003	-0.073	-.28**	-.19**	.37**	.33**	0.09	.18**	0.001	.26**	0.04	.58**	-.21**	__		
20	Physical symptoms in the past six months	-0.12	-0.04	0.05	0.003	-.12*	0.06	0.04	-.15*	-0.06	.39**	.27**	0.09	0.07	-0.11	.19**	.26**	.80**	-0.10	.66**	__	
21	Well-being in the last six months	.27**	.13*	0.12	.15*	.15*	.18**	.18**	.29**	.32**	-.14*	-.22**	0.02	.15*	.13*	.21**	.19**	0.02	.57**	0.07	.1**	__

*$p < .05$; **$p < .01$.

Table 4

Scores of the variables included in the regression analyses.

	Items	Score	Items	Scale score	Minimum and maximum scores
Step 1					
Constant					
Gender	Men<br>Women	0-1			
Age		1-19			
Ethnicity	Mestizo<br>0thers	0-1			
Family Condition	Nuclear<br>Others	0-1			
Marital status	Single<br>Others	0-1			
Currently in a relationship	No<br>Yes	0-1			
Satisfaction with studies	Do not know<br>Very low<br>Low<br>Medium<br>High	1,2,3,4,5			
Works	No<br>Yes	0-1			
Dependent persons	No<br>Yes	0-1			
Affiliated with health services	No<br>Yes	0-1			
Chronic health problem	No<br>Yes	0-1			
Treatment effectiveness: health problems	Not applicable<br>No<br>Yes	0,1,2			
Functional diversity	No<br>Yes	0-1			
Treatment or rehabilitation	Not applicable<br>No<br>Yes	0,1,2			
Physical violence	No<br>Yes	0-1			
Sexual violence	No<br>Yes	0-1			
Psychological violence	No<br>Yes	0-1			
Social network aggression	No<br>Yes	0-1			
Forced displacement	No<br>Yes	0-1			
Step 2					
Constant					
Well-being at home			4	0-6	0-24
Well-being in the neighborhood			4	0-6	0-24
Well-being in religious organizations			4	0-6	0-24
Social welfare, culture and recreation			4	0-6	0-24
School wellness			4	0-6	0-24
Well-being at work			4	0-6	0-24
Expectation of social support			8	0-6	0-48
Healthy lifestyle habits			8	1-6	1-48
Psychosocial skills for life			10	1-5	10-50
Worry and emotional distress			5	0-4	0-20
Suicidal behavior			3	0-1	0-3
Drug use			5	0-4	0-20
Conflicts due to drug use			7	0-2	0-14

Problematic internet use			4	0-4	0-16
Problems due to problematic internet use			7	0-2	0-14
Emotional symptoms in the past six months			8	1-5	8-40
Well-being in the last six months			4	1-4	4-20
Diagnosis in mental health					
In treatment: mental health					

Prediction of Emotional Symptoms in the Last Six Months

Participants without a mental health diagnosis (n= 980)

A hierarchical regression analysis was performed using a sample of 980 participants without a mental health diagnosis, aiming to predict negative emotional symptoms and psychological distress experienced over the past six months, based on well-being across different environments and various risk and protective factors (Table 4). In the first step, structural variables were included, and the model was statistically significant, explaining 13% of the variance ($R^2 = .13$; $F_{(19, 961)} = 7.333$, $p < .001$). Variables strongly associated with emotional symptoms included being in a romantic relationship ($\beta = –.27$, $t = –2.02$, p = .006, 95% CI [–17.37, –0.28]), which was linked to lower emotional symptomatology; lower satisfaction with academic life ($\beta = –.14$, $t = –4.63$, $p < .001$, 95% CI [–1.52, –0.61]); and having experienced physical ($\beta = .17$, $t = 5.46$, $p < .001$, 95% CI [2.78, 5.89]), sexual ($\beta = .13$, $t = 4.27$, $p < .001$, 95% CI [2.34, 6.31]), and psychological violence ($\beta = .24$, $t = 7.65$, $p < .001$, 95% CI [3.94, 6.66]), all of which were associated with higher levels of emotional symptoms. Additionally, a significant relationship was found between emotional symptoms and having experienced aggression on social media ($\beta = .09$, $t = 2.87$, p = .011, 95% CI [1.85, 9.80]).

In the second step, intermediate factors were included, enhancing the model's explanatory power, accounting for 29% of the variance ($R^2 = .29$; $F_{(34, 961)} = 11.174$, p < .001), with a significant increase of $\Delta R^2 = .16$. The variables that contributed most to this explanation were lower levels of healthy lifestyle habits ($\beta = –.15$, $t = –4.45$, $p < .001$, 95% CI [–0.30, –0.12]), higher levels of worry and emotional distress ($\beta = .21$, t = 6.69, p < .001, 95% CI [0.34, 0.62]), greater suicidal behavior ($\beta = .20$, $t = 6.53$, $p < .001$, 95% CI [1.20, 2.23]), and problems due to problematic internet use ($\beta = .15$, $t = 4.47$, $p < .001$, 95% CI [0.24, 0.61]). Other variables, such as drug use ($\beta = .06$, $t = 1.98$, p = .048, 95% CI [0.002, 0.41]) and related conflicts ($\beta = –.07$, $t = –1.94$, p = .052, 95% CI [–0.32, 0.00]), revealed marginal yet notable effects. The full results of all reported regression analyses can be found in Supplementary Material 1.

Table 5

Results of the multiple regression predicting emotional symptoms and distress over the last six months.

	Participants without a mental health diagnosis (n= 980)							
Variables	B	95% CI para-B ---------------------- - LI	LS	SE B	t	β	R^2	ΔR^2
Step 1								
Constant	19.94***	12.719	27.169	3.682	5.417		.13	.13
Gender	-.004	-.996	.989	.506	-.007	.000		
Age	-.026	-.242	.190	.110	-.236	-.007		
Ethnicity	-.773	-1.883	.336	.565	-1.368	-.04		
Family Condition	-.859	-3.246	1.529	1.217	-.706	-.02		
Marital status	3.758	-.499	8.014	2.169	1.732	.23		
Currently in a romantic relationship	-8.827	-17.374	-.279	4.355	-2.027	-.27*		
Satisfaction with studies	-1.064	-1.515	-.613	.230	-4.629	-.14***		
Works	-.602	-1.755	.552	.588	-1.023	-.03		
Dependent persons	.725	-.228	1.679	.486	1.492	.05		
Affiliated with health services	3.053	.620	5.487	1.240	2.463	.07*		
Chronic health problem	-2.705	-11.778	6.368	4.623	-.585	-.10		
Treatment effectiveness: health problems	1.202	-2.164	4.569	1.715	.701	.12		

Functional diversity	.484	-3.182	4.150	1.868	.259	.02		
Treatment or rehabilitation	.277	-1.928	2.482	1.123	.247	.02		
Physical violence	4.332	2.776	5.888	.793	5.464	.17***		
Sexual violence	4.322	2.337	6.306	1.011	4.273	.13***		
Psychological violence	5.304	3.944	6.664	.693	7.654	.24***		
Social network aggression	5.824	1.846	9.801	2.027	2.873	.09**		
Forced displacement	1.898	-.698	4.494	1.323	1.435	.04		
Step 2								
Constant	17.727***	10.150	25.305	3.861	4.591		.29	.16
Well-being at home	-.109	-.251	.034	.073	-1.491	-.05		
Well-being in the neighborhood	.026	-.084	.135	.056	.461	.01		
Well-being in religious organizations	.045	-.020	.110	.033	1.356	.04		
Social welfare, culture and recreation	-.009	-.069	.051	.031	-.285	-.009		
School wellness	-.062	-.205	.080	.073	-.857	-.03		
Well-being at work	.056	-.049	.160	.053	1.046	.05		
Expectation of social support	.012	-.045	.069	.029	.411	.01		
Healthy lifestyle habits	-.206	-.297	-.115	.046	-4.453	-.15***		
Psychosocial skills for life	.007	-.059	.073	.034	.212	.007		
Worry and emotional distress	.476	.337	.616	.071	6.694	.21***		
Suicidal behavior	1.715	1.199	2.231	.263	6.526	.20***		
Drug use	.207	.002	.412	.104	1.983	.06*		
Conflicts due to drug use	-.159	-.319	.001	.082	-1.945	-.07*		
Problematic internet use	-.107	-.289	.075	.093	-1.157	-.03		
Problems due to problematic internet use	.424	.238	.611	.095	4.470	.15***		

Note. B = unstandardized coefficient; CI = confidence interval; LL = lower limit; UL = upper limit; *t* = test of variance; SE-B = standard error of B; β = standardized coefficient.
*p < .05. **p < .01. ***p < .001.

Prediction of Emotional Symptoms in the Last Six Months

Participants with a current diagnosis of mental health problems (n= 252)

A second hierarchical regression analysis was conducted to predict emotional symptoms and psychological distress experienced over the past six months by 252 participants with a current mental health diagnosis. In the first step, structural variables were included, and the model was statistically significant, explaining 20% of the variance ($R^2 = .20$; $F(19, 250) = 3.058$, $p < .001$). Among the variables significantly strongly associated with higher emotional symptoms were included having experienced physical violence ($\beta = .29$, $t = 4.49$, $p < .001$, 95% CI [3.56, 9.12]) and psychological violence ($\beta = .26$, $t = 3.88$, $p < .001$, 95% CI [2.35, 7.20]).

In the second step, intermediate factors were added, significantly increasing the explained variance to 38% ($R^2 = .38$; $F_{(34, 961)} = 3.874$, $p < .001$), with a ΔR^2 increase of .18. Worry and emotional distress ($\beta = .28$, $t = 4.37$, $p < .001$, 95% CI [0.35, 0.92]) along with problems due to problematic internet use ($\beta = .18$, $t = 2.65$, $p = .009$, 95% CI [0.14, 0.96]) were key contributors to the model.
The full results are available in Supplementary Material 1.

Table 6

Results of the multiple regression predicting emotional symptoms and distress over the last six months.

	Participants with a current diagnosis of mental health problems (n= 252)						
Variables	B	95% CI para-B	SE B	t	β	R^2	ΔR^2

		LI	LS					
Step 1								
Constant	17.046***	2.901	31.191	7.179	2.374		.20	.20
Gender	1.948	-.102	3.998	1.040	1.873	.11		
Age	.260	-.191	.710	.229	1.136	.06		
Ethnicity	-1.411	-3.823	1.001	1.224	-1.153	-.07		
Family Condition	2.686	-1.970	7.343	2.363	1.137	.07		
Marital status	1.414	-7.042	9.871	4.292	.330	.073		
Currently in a relationship	-4.849	-19.731	10.032	7.553	-.642	-.14		
Satisfaction with studies	-.789	-1.642	.064	.433	-1.821	-.11		
Works	.399	-1.931	2.729	1.183	.338	.02		
Dependent persons	-2.402	-4.825	.021	1.230	-1.953	-.13		
Affiliated with health services	2.907	-2.026	7.840	2.504	1.161	.07		
Chronic health problem	-1.009	-15.568	13.551	7.390	-.136	-.04		
Treatment effectiveness: health problems	.769	-4.437	5.975	2.642	.291	.10		
Functional diversity	3.565	-3.774	10.903	3.725	.957	.16		
Treatment or rehabilitation	-1.352	-5.851	3.147	2.283	-.592	-.10		
Physical violence	6.343	3.563	9.124	1.411	4.494	.29***		
Sexual violence	1.537	-1.651	4.724	1.618	.950	.06		
Psychological violence	4.776	2.352	7.199	1.230	3.883	.26***		
Social network aggression	5.895	-3.127	14.918	4.579	1.287	.07		
Forced displacement	.332	-8.782	9.445	4.626	.072	.004		
Step 2								
Constant	11.123	-4.093	26.338	7.720	1.441		.38	.18
Well-being at home	.096	-.174	.367	.137	.702	.04		
Well-being in the neighborhood	-.164	-.365	.036	.102	-1.614	-.10		
Well-being in religious organizations	-.102	-.262	.059	.081	-1.251	-.08		
Social welfare, culture and recreation	-.024	-.149	.100	.063	-.387	-.02		
School wellness	-.096	-.412	.220	.160	-.599	-.04		
Well-being at work	.203	-.061	.468	.134	1.517	.19		
Expectation of social support	.047	-.071	.166	.060	.789	.05		
Healthy lifestyle habits	-.186	-.384	.012	.100	-1.855	-.14		
Psychosocial skills for life	.093	-.037	.223	.066	1.404	.09		
Worry and emotional distress	.636	.349	.924	.146	4.367	.28***		
Suicidal behavior	.849	-.044	1.742	.453	1.874	.12		
Drug use	.086	-.263	.435	.177	.485	.03		
Conflicts due to drug use	.026	-.303	.355	.167	.155	.01		
Problematic internet use	.180	-.182	.542	.184	.982	.05		
Problems due to problematic internet use	.547	.140	.955	.207	2.650	.18**		

Note. B = unstandardized coefficient; CI = confidence interval; LL = lower limit; UL = upper limit; *t* = test of variance; SE-B = standard error of B; β = standardized coefficient.

*p < .05. **p < .01. ***p < .001.

Prediction of Well-being in the Last Six Months

Participants without a mental health diagnosis (n= 980)

A third hierarchical regression analysis was conducted performed with using a sample of 980 participants without a mental health diagnosis, aiming to predict emotional well-being over the past six months based on structural and intermediate variables. In the first step, structural variables were included, and although the model was statistically significant, it showed low explanatory power, accounting for 5% of the variance ($R^2 = .05$; F (19, 961) = 1.391, $p < .001$). Within this block, the only variables that showed significant associations were ethnic group (β = –.08, t = –2.31, p = .021, 95% CI [–1.18, –0.10]), indicating lower well-being among participants belonging to ethnic minority groups, and satisfaction with academic life (β = .10, t = 3.39, $p < .001$, 95% CI [0.16, 0.60]), which was positively associated with well-being.

In the second step, intermediate factors were included, and the model's explanatory capacity improved, reaching 13% of the variance ($R^2 = .13$; $F_{(34, 961)} = 2.990$, $p < .001$), with a significant increase of $\Delta R^2 = .08$. The variables that contributed most significantly to the prediction of well-being were psychosocial life skills (β = .22, *t* = 6.28, $p < .001$, 95% CI [0.08, 0.14]), expected social support (β = .08, *t* = 2.06, p = .039, 95% CI [0.002, 0.06]), access to sociocultural and recreational well-being (β = –.09, *t* = –2.37, *p* = .018, 95% CI [–0.07, –0.01]), and lower well-being at work (β = –.12, *t* = –2.04, p = .042, 95% CI [–0.11, –0.002]).

Table 7

Results of the multiple regression predicting emotional well-being over the last six months.

	Participants with a current diagnosis of mental health problems (n= 980)							
Variables	B	95% CI para-B		SE B	t	β	R^2	ΔR^2
		LI	LS					
Step 1								
Constant	10.379***	6.847	13.911	1.800	5.766		.05	.05
Gender	-.139	-.624	.346	.247	-.562	-.018		
Age	-.045	-.150	.061	.054	-.832	-.027		
Ethnicity	-.641	-1.183	-.098	.276	-2.318	-.076*		
Family Condition	.170	-.997	1.338	.595	.287	.009		
Marital status	1.261	-.821	3.342	1.060	1.189	.163		
Currently in a relationship	-3.418	-7.598	.761	2.129	-1.605	-.220		
Satisfaction with studies	.381	.161	.602	.112	3.394	.109***		
Works	.070	-.494	.634	.287	.243	.008		
Dependent persons	-.330	-.796	.136	.238	-1.388	-.049		
Affiliated with health services	.834	-.356	2.023	.606	1.375	.044		
Chronic health problem	-3.081	-7.517	1.355	2.260	-1.363	-.236		
Treatment effectiveness: health problems	.698	-.948	2.344	.839	.832	.144		
Functional diversity	-.615	-2.407	1.177	.913	-.673	-.064		
Treatment or rehabilitation	.152	-.926	1.229	.549	.276	.026		
Physical violence	-.317	-1.078	.443	.388	-.819	-.027		
Sexual violence	.388	-.582	1.358	.494	.785	.026		
Psychological violence	.562	-.103	1.227	.339	1.659	.055		
Social network aggression	1.799	-.146	3.743	.991	1.815	.058		
Forced displacement	-.054	-1.323	1.216	.647	-.083	-.003		
Step 2								

Constant	3.841*	-.084	7.767	2.000	1.921	-.018	.13	.08
Well-being at home	.070	-.004	.144	.038	1.849	.066		
Well-being in the neighborhood	-.003	-.060	.053	.029	-.114	-.004		
Well-being in religious organizations	.003	-.030	.037	.017	.197	.007		
Social welfare, culture and recreation	-.038	-.069	-.006	.016	-2.368	-.086*		
School wellness	.055	-.018	.129	.038	1.473	.052		
Well-being at work	-.056	-.110	-.002	.028	-2.035	-.120*		
Expectation of social support	.031	.002	.061	.015	2.064	.075*		
Healthy lifestyle habits	.022	-.025	.069	.024	.918	.034		
Psychosocial skills for life	.109	.075	.143	.017	6.287	.221***		
Worry and emotional distress	.039	-.033	.111	.037	1.061	.037		
Suicidal behavior	-.080	-.347	.187	.136	-.589	-.020		
Drug use	-.058	-.165	.048	.054	-1.081	-.038		
Conflicts due to drug use	.034	-.049	.117	.042	.807	.032		
Problematic internet use	.056	-.038	.150	.048	1.169	.040		
Problems due to problematic internet use	.081	-.016	.177	.049	1.642	.062		

Note. B = unstandardized coefficient; CI = confidence interval; LL = lower limit; UL = upper limit; *t* = test of variance; SE-B = standard error of B; β = standardized coefficient.
*p < .05. **p < .01. ***p < .001.

Prediction of Well-being in the Last Six Months

Participants with a current diagnosis of mental health problems (n= 252)

Finally, a hierarchical regression analysis was conducted to predict emotional well-being over the past six months in a sample of 252 participants with a current mental health diagnosis. In the first step, structural variables were included. The model explained 10% of the variance (R^2 = .10; $F_{(19, 250)}$ = 1.391, p = .132), although it was not statistically significant. The only variables that showed significant associations with higher well-being were satisfaction with academic life (β = .20, *t* = 3.00, p = .003, 95% CI [0.22, 1.07]) and affiliation with healthcare services (β = .14, *t* = 2.07, p = .039, 95% CI [0.13, 5.04]). Other structural variables did not show statistically significant effects.

In the second step, intermediate factors were incorporated, significantly improving the model, which explained 32% of the variance (R^2 = .32; $F_{(34, 961)}$ = 2.990, *p* < .001), with an increase of ΔR^2 = .22. Variables significantly associated with higher levels of well-being included well-being at home (β = .23, *t* = 3.31, *p* < .001, 95% CI [0.09, 0.36]), well-being at work (β = .34, *t* = 2.49, p = .013, 95% CI [0.03, 0.29]), and psychosocial life skills (β = .23, *t* = 3.29, *p* < .001, 95% CI [0.04, 0.17]). On the other hand, suicidal behavior was negatively associated with well-being (β = –.18, *t* = –2.65, p = .009, 95% CI [–1.03, –0.15]), as were problems due to problematic internet use (β = –.26, *t* = –3.58, *p* < .001, 95% CI [0.16, 0.56]), indicating an adverse impact.

Table 8

Results of the multiple regression predicting emotional well-being over the last six months.

	Participants with a current diagnosis of mental health problems (n= 252)							
Variables	B	95% CI para-B		SE B	t	β	R^2	ΔR^2
		LI	LS					
Step 1								
Constant	9.628*	2.589	16.666	3.572	2.695		.10	.10
Gender	-.243	-1.263	.777	.518	-.470	-.031		
Age	.047	-.177	.271	.114	.413	.026		
Ethnicity	-.407	-1.608	.793	.609	-.669	-.043		
Family Condition	.928	-1.389	3.245	1.176	.789	.053		
Marital status	-3.430	-7.638	.778	2.136	-1.606	-.379		
Currently in a relationship	5.391	-2.014	12.796	3.758	1.434	.339		
Satisfaction with studies	.647	.222	1.071	.215	3.000	.199*		
Works	.967	-.193	2.126	.588	1.643	.117		
Dependent persons	.138	-1.067	1.344	.612	.226	.016		
Affiliated with health services	2.583	.129	5.038	1.246	2.074	.136*		
Chronic health problem	-6.375	-13.620	.870	3.677	-1.734	-.666		
Treatment effectiveness: health problems	2.177	-.414	4.767	1.315	1.656	.639		
Functional diversity	-.831	-4.482	2.821	1.853	-.448	-.081		
Treatment or rehabilitation	.210	-2.029	2.448	1.136	.185	.033		
Physical violence	-.672	-2.056	.712	.702	-.957	-.068		
Sexual violence	-.434	-2.019	1.152	.805	-.539	-.037		
Psychological violence	.145	-1.061	1.351	.612	.237	.017		
Social network aggression	1.340	-3.150	5.829	2.279	.588	.038		
Forced displacement	-.055	-4.590	4.480	2.302	-.024	-.002		
Step 2								
Constant	2.726	-4.748	10.201	3.792	.719		.32	.22
Well-being at home	.223	.091	.356	.067	3.314	.230***		
Well-being in the neighborhood	-.038	-.137	.060	.050	-.764	-.051		
Well-being in religious organizations	-.004	-.083	.075	.040	-.103	-.007		
Social welfare, culture and recreation	-.005	-.066	.056	.031	-.175	-.012		
School wellness	-.037	-.192	.118	.079	-.468	-.034		
Well-being at work	.164	.034	.294	.066	2.494	.337*		
Expectation of social support	.012	-.047	.070	.030	.398	.027		
Healthy lifestyle habits	-.012	-.109	.085	.049	-.239	-.019		
Psychosocial skills for life	.107	.043	.171	.032	3.293	.231***		
Worry and emotional distress	.017	-.124	.158	.072	.237	.016		
Suicidal behavior	-.591	-1.029	-.152	.223	-2.653	-.178**		
Drug use	-.089	-.260	.083	.087	-1.018	-.069		

Conflicts due to drug use	.052	-.110	.213	.082	.632	.049
Problematic internet use	.096	-.082	.274	.090	1.062	.067
Problems due to problematic internet use	.363	.163	.563	.101	3.576	.257***

Note. B = unstandardized coefficient; CI = confidence interval; LL = lower limit; UL = upper limit; *t* = test of variance; SE-B = standard error of B; β = standardized coefficient.
*p < .05. **p < .01. ***p < .001.

Prediction of emotional well-being in the last six months (full sample)

A hierarchical regression was performed to investigate whether well-being in different settings and risk and protective factors (intermediate factors in the questionnaire) predicted emotional well-being in the last six months. Gender, age, and factors representing these components were included in the analysis.

The model evaluated was significant ($R^2 = .12$; $\Delta R^2 = .12$). The variables that contributed to the variance explained were well-being at home ($\beta = .10$; $t = 3.291$, $p < .001$; 95% CI = 0.04, 0.16); school well-being ($\beta = .06$; $t = 2.247$, $p < .05$; 95% CI = 0.009, 0.13), where higher well-being in the school environment predicted better general well-being; and social well-being, culture and leisure ($\beta = -.06$; $t = -1.992$, $p < .05$; 95% CI = -0.05, 0. 000), where lower well-being in these areas negatively influenced general well-being. Also contributing to the variance explained were the expectation of social support ($\beta = 0.06$; $t = 1.930$, $p < .05$; 95% CI = 0.000, 0.05), psychosocial life skills ($\beta = 0.21$; $t = 6.987$, $p < .001$; 95% CI = 0.07 to 0.13), which emerged as the most significant positive predictor of well-being, and problems due to problematic internet use ($\beta = 0.08$; $t = 2.744$, $p < .05$; 95% CI = 0.03 to 0.20).

We replicated the regression analysis by differentiating participants with a mental health diagnosis (n = 252) from those without one (n = 980). The variables affecting emotional well-being in the last six months were consistent across the entire group and these subgroups. The variables that showed the greatest effect on well-being were well-being at home and psychosocial life skills. The results of the regression are shown in the following table.

Table 9

Results of the multiple regression predicting well-being in the last six months (N=1232).

Variables	B	95% CI para *B*		SE B	t	β	R^2	ΔR^2
		LI	LS					
							.12	.12
Constant	4.40***	1.768	7.048	1.34	3.276			
Gender	-.11	-.524	.303	.21	-.524	-.01		
Age	-.02	-.118	.063	.04	-.600	-.01		
Well-being at home	.10	.042	.168	.03	3.291	.10***		
Well-being in the neighborhood	-.02	-.068	.029	.02	-.798	-.02		
School wellness	.07	.009	.136	.03	2.247	.06*		
Well-being at work	-.008	-.035	.019	.014	-.559	-.01		
Well-being in religious organizations	-.003	-.033	.027	.015	-.199	-.006		
Social welfare, culture and recreation	-.027	-.054	.000	.014	-1.992	-.06*		
Expectation of social support	.025	.000	.051	.013	1.930	.06*		
Healthy lifestyle habits	.026	-.015	.066	.02	1.225	.04		
Psychosocial skills for life	.10	.074	.132	.01	6.987	.21***		

Worry and emotional distress	.029	-.033	.091	.03	.914	.02
Suicidal behavior	-.14	-.354	.064	.10	-1.363	-.03
Drug use	-.08	-.170	.005	.04	-1.843	-.05
Conflicts due to drug use	.05	-.019	.125	.03	1.441	.04
Problematic internet use	.08	.000	.162	.041	1.961	.05
Problems due to problematic internet use	.11	.034	.203	.043	2.744	.08*

Note. B = unstandardized coefficient; CI = confidence interval; LL = lower limit; UL = upper limit; *t* = test of variance; SE-B = standard error of B; β = standardized coefficient.

*p < .05. **p < .01. ***p < .001.
